# De Dissectione Synchondroseos Ossium Pubis in partu difficili

**Published:** 1782

**Authors:** 


IV. Joann. Gottlieb Walter, M. D. Anatom. Profeff. &c. *De Diffectione Synchondrofeos Offium Pubis in partu difficili.* 4to. Berlin, 1782. p. 32, cum figurâ.

THIS ingenious profeffor is the firft writer on the Continent who has ventured to difpute pute the utility and propriety of the section of the symphysis pubis, in cases of narrow pelvis. Hitherto we have read only of the ease and success with which it has been performed in different countries, but now that the novelty of the thing is in some measure subsided, and practitioners begin to consider it more coolly and candidly, we may hope to see the subject impartially discussed. If in this country we have been less sanguine than the rest of Europe in our ideas on this point, we owe our caution and moderation in a great measure to Dr. Hunter, who very early pointed out the difficulties, dangers, and disadvantages which might be objected to the operation, and put us upon our guard against them.

The author of the work before us derives the greater part of his arguments from anatomy. He observes, that the union of the ossa pubis is not (as some have asserted) effected by means of a common cartilage and an annular ligament, but that each of these two bones has its proper cartilage, and that the junction is by means of an intermediate ligament. If the section is performed, the middle of the symphysis, he tells us, must be accurately fixed on, to avoid wounding the cartilages, the cure of which might be very tedious, and attended with many inconveniencies.

He

He is persuaded, likewise, that even if the intermediate ligament only were to be divided, the cure, as in other wounds of the ligaments, would be very difficult.

He controverts the opinion, that the ligaments are moistened and relaxed by an afflux of humours towards the pelvis, in the advanced stage of pregnancy. He asserts, that he has dissected more than an hundred women, who died a little before or soon after delivery, and never yet found that change in the consistence of the cartilages and ligaments, which the advocates for the section so uniformly mention.

He is convinced that in cases of narrow pelvis, the space gained by slitting the symphysis pubis will not be sufficient to allow a large head to pass. In such instances, therefore, he gives the preference to the Cæsarean operation, which he advises to be performed at the linea alba.

In the course of his work, Dr. Walter takes occasion to describe a male pelvis, of which an engraving is given, in which the ossa pubis were two inches and a half distant from each other, and held together only by means of the transverse ligament, altho' the sacro-iliac ligaments were in a natural state.

